# Nectin-4 Expression in Muscle-Invasive Bladder Cancer Is Associated with Growth-Related and Inflammatory Signaling Pathways

**DOI:** 10.3390/ijms27135706

**Published:** 2026-06-24

**Authors:** Sebastian Jersinovic, Marko Vukovic, Jörg Hennenlotter, Thomas Lütfrenk, Tilman Todenhöfer, Arnulf Stenzl, Igor Tsaur, Steffen Rausch

**Affiliations:** 1 Department of Urology, Eberhard-Karls-University, 72074 Tuebingen, Germanyhennenlotter-eningen@freenet.de (J.H.); thomas.luetfrenk@med.uni-tuebingen.de (T.L.); eau@stenzl.net (A.S.); urologie@med.uni-tuebingen.de (I.T.); steffen.rausch@med.uni-tuebingen.de (S.R.); 2 Center of Modern Urology, 1000 Ljubljana, Slovenia; 3 Department of Urology, Clinical Centre of Montenegro, University of Montenegro, 81000 Podgorica, Montenegro; 4 Clinical Practice in Urology, 72622 Nuertingen, Germany; todenhoefer@studienurologie.de

**Keywords:** urothelial cancer, prognosis, biomarker, Nectin-4

## Abstract

Nectin-4 has emerged as a clinically relevant target in muscle-invasive bladder cancer (MIBC), primarily because of its role in antibody–drug conjugate-based therapies. However, the broader biological context of Nectin-4 expression and its association with tumor-promoting signaling pathways in MIBC remain insufficiently characterized. In this single-institution study, Nectin-4 expression (H-score 0–300) was assessed by immunohistochemistry in two independent MIBC cohorts. Associations between Nectin-4 expression and key markers related to growth signaling, metabolic regulation, and inflammation were analyzed alongside clinicopathological characteristics. Nectin-4 expression was significantly higher in malignant tissue than in non-malignant tissue (*p* = 0.0016 and *p* = 0.0302, respectively). Nectin-4 expression was not associated with demographic or clinicopathological parameters; however, a trend toward lower expression in more advanced disease stages was observed. Significant positive correlations were identified between Nectin-4 expression and protein kinase B (*p* = 0.0004), cytoplasmic (*p* = 0.0115) and membranous somatostatin receptor 2 (*p* = 0.0125), insulin receptor substrate 1 (*p* = 0.03), and interleukin-1 receptor antagonist (IL-1RA; *p* = 0.0045). In contrast, a negative correlation was observed with the IL-1β/IL-1RA ratio (*p* = 0.0246). Although Nectin-4 expression was not significantly associated with cancer-specific or overall survival, a trend toward shorter relapse-free survival was observed in patients with lower Nectin-4 expression (*p* = 0.0531). In multivariate analysis, patient age, but not Nectin-4 expression, emerged as an independent prognostic factor. Although Nectin-4 expression does not appear to have independent prognostic value, its biological associations suggest that it reflects an integrated tumor-related signaling context. These findings support further investigation of Nectin-4 as part of rational, biology-driven therapeutic strategies in bladder cancer.

## 1. Introduction

Bladder cancer (BC) remains a common malignancy associated with substantial morbidity and mortality worldwide [1]. The identification of reliable biomarkers for prognosis and therapeutic targeting is therefore essential to improve patient management and clinical outcomes [2]. Nectin-4, a member of the nectin family of cell adhesion molecules, has recently gained considerable attention because of its involvement in tumor cell adhesion, migration, and signaling pathways across various malignancies [3,4].

In locally advanced or metastatic urothelial carcinoma, the phase III EV-302 trial (also known as KEYNOTE-A39) demonstrated that the combination of the Nectin-4-targeted antibody–drug conjugate Enfortumab Vedotin and Pembrolizumab significantly improved progression-free survival (PFS) and overall survival (OS) compared with platinum-based chemotherapy. In this randomized study of previously untreated patients, median PFS nearly doubled, while median OS exceeded 30 months, representing a major advance over historical standards of care [5].

More recently, in the muscle-invasive bladder cancer (MIBC) setting, the phase III KEYNOTE-905/EV-303 trial demonstrated that perioperative treatment with Enfortumab Vedotin plus Pembrolizumab, administered before and after radical cystectomy, significantly improved event-free survival (EFS) and OS compared with surgery alone in patients who were either ineligible for or declined cisplatin-based chemotherapy. The study further reported higher rates of pathological complete response and substantial reductions in the risk of disease progression or death, ultimately leading to regulatory approval of this combination for perioperative treatment of MIBC [6].

Previous studies have demonstrated Nectin-4 expression in urothelial carcinoma and suggested associations with tumor aggressiveness [7,8,9]. However, comprehensive immunohistochemical analyses correlating Nectin-4 expression with clinicopathological characteristics and survival outcomes in BC remain limited [3,9]. Klümper et al. further proposed Nectin-4 staining intensity as a potential prognostic and predictive biomarker in metastatic BC [9]. Given the rapidly evolving role of Enfortumab Vedotin in earlier disease stages, additional insight into the biological significance and functional context of Nectin-4 in BC is of considerable interest.

Beyond its established role as a therapeutic target and predictive marker for antibody–drug conjugate therapy, Nectin-4 represents a biologically relevant adhesion molecule that may be integrated into broader signaling networks involved in tumor progression. Emerging evidence suggests that Nectin-4 expression is associated with pathways related to cell growth, metabolic regulation, and tumor-associated inflammation [9]. In the context of MIBC, where perioperative treatment strategies are rapidly evolving, a deeper understanding of Nectin-4-associated biology may contribute to the development of rational combination therapies and optimized neoadjuvant treatment concepts.

The investigated signaling markers were selected based on their established or proposed roles in tumor progression, growth signaling, metabolic regulation, and inflammation in bladder cancer and other solid malignancies. Protein kinase B (Akt) and insulin receptor substrate 1 (IRS-1) represent key components of proliferative and metabolic signaling pathways, while somatostatin receptor 2 (SSTR2) has been implicated in tumor differentiation and receptor-mediated signaling. In addition, inflammatory mediators including IL-1 receptor antagonist (IL-1RA) and the IL-1β/IL-1RA ratio were included to evaluate the relationship between Nectin-4 expression and the inflammatory tumor microenvironment. We hypothesized that Nectin-4 expression may reflect a broader biologically integrated signaling context associated with tumor-promoting processes in MIBC.

The aim of the present study was, therefore, not only to characterize Nectin-4 expression patterns in MIBC, but also to explore its associations with key signaling and inflammatory markers, namely somatostatin receptor 2 (SSTR2), insulin receptor substrate 1 (IRS1), Protein kinase B (AKT) and interleukin-1 beta (IL-1β) (including the Il-1ß/IL-receptor antagonist (IL-RA) axis) that may provide biological context and translational relevance for future perioperative treatment strategies [4,7,8,10].

## 2. Results

### 2.1. Patients’ Characteristics

Patients were divided into two cohorts based on previously reported associations with inflammatory and oncologic biomarkers. The discovery cohort comprised 103 patients, including 76.7% men, with a median age of 69 years (range, 32–84 years). Analyses investigating correlations with inflammatory and oncologic biomarkers were performed exclusively in this cohort. Most tumors were high grade (G3, 76.5%). Lymph node status was distributed as follows: N0 in 58.2% and N1–N3 in 41.8% of cases. The majority of patients had no distant metastases (M0, 90.1%). Complete tumor resection (R0) was achieved in 81.4% of patients. The validation cohort included 97 patients, 72.2% of whom were male, with a median age of 65.5 years (range, 40–83 years). Tumor stages were distributed as T1–T2b in 32%, T3a–T3b in 45%, and T4a–T4b in 23% of patients. No statistically significant differences in baseline clinicopathological characteristics were observed between the two cohorts, indicating that the groups were well balanced (Table 1). No patients received neoadjuvant treatment.

### 2.2. Expression Characteristics of Nectin-4

**Figure 1:**
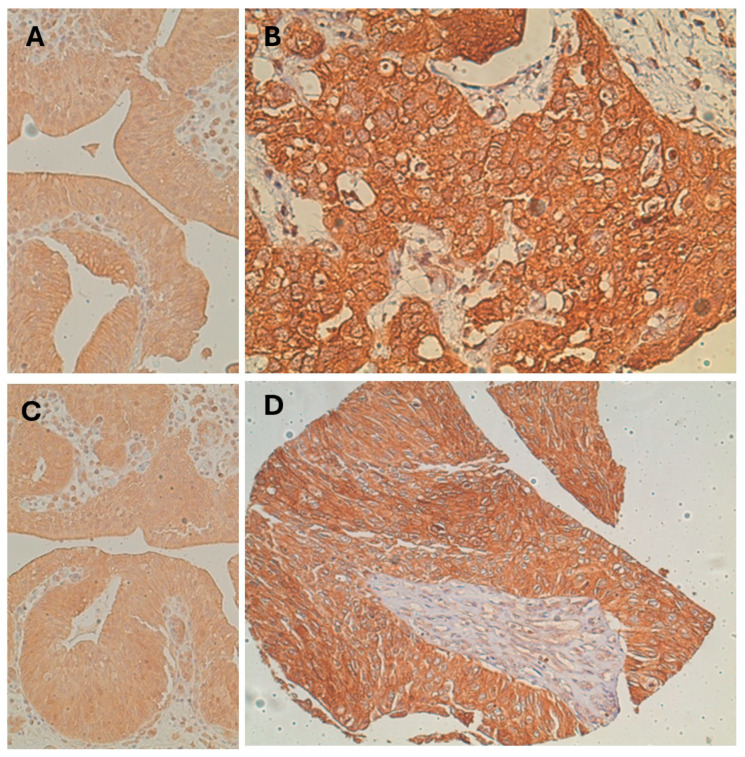
(**A**–**D**): Representative staining for immunohistochemistry of Nectin-4 in bladder tumor and benign tissues. (**A**)—Staining in benign tissue; (**B**)—High membranous and cytoplasmatic staining in cancer tissue within first group; (**C**)—Staining in benign tissue; (**D**)—High membranous and cytoplasmatic staining in cancer tissue within validation cohort.

**Figure 2:**
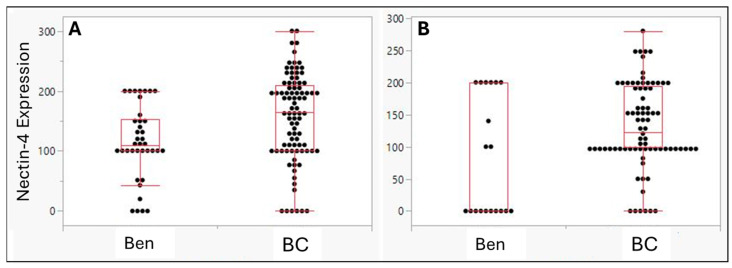
(**A**,**B**): Single factor analysis of Nectin-4 expression in benign urothelium (Ben) and urothelial carcinoma tissue (BC) of (**A**): The discovery group and (**B**): The validation cohort (mean ± SD: 114 ± 10 vs. 158 ± 8 and 81 ± 21 vs. 133 ± 8; *p* = 0.0016 and 0.0302, respectively).

This evaluation referred to the overall immunohistochemical expression of Nectin-4 in both tumor and non-malignant tissues. In general, staining was predominantly observed in epithelial cells, particularly within the urothelium. The staining pattern extended uniformly from the basal cell layer to the apically located luminal cell layer. Tumor cells exhibited either a diffuse staining pattern or appeared in large clustered aggregates. The median H-scores of urothelial carcinoma were 165 and 123 in the two cohorts, respectively (*p* = 0.5). In both cohorts, tumor tissue demonstrated significantly higher staining intensity compared with the corresponding normal (benign) urothelium (H-scores: 165 vs. 140, *p* = 0.0016; and 123 vs. 90, *p* = 0.0302, respectively) (Figure 1 and Figure 2).

### 2.3. Expression Distribution in Bladder Cancer Samples

**Figure 3:**
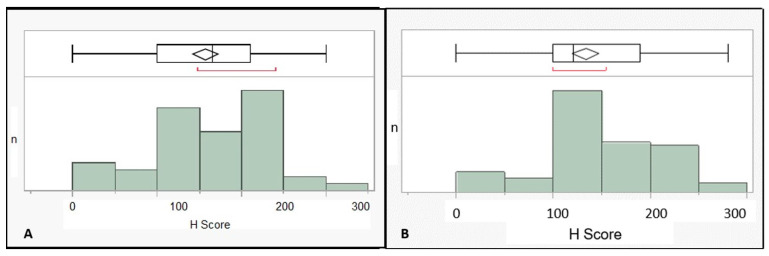
(**A**,**B**): Distributions of individual expressions in bladder cancer tissues from (**A**): The discovery cohort and (**B**): The validation cohort.

The expression distributions shown in Figure 3A,B demonstrate consistent Nectin-4 expression patterns across both cohorts, with comparable ranges and quartile distributions (discovery cohort: median 165, IQR 100–210, range 0–300; validation cohort: median 123, IQR 100–195, range 0–280).

### 2.4. Correlations with Clinicopathological Parameters

No significant associations were observed between Nectin-4 expression and gender, T stage, lymph node status, metastatic status, tumor grade, or the presence of carcinoma in situ (all *p* > 0.1). Nevertheless, a non-significant trend toward lower Nectin-4 expression was noted in tumors with lymph node involvement and in high-grade (G3) tumors.

### 2.5. Survival Analysis

**Figure 4:**
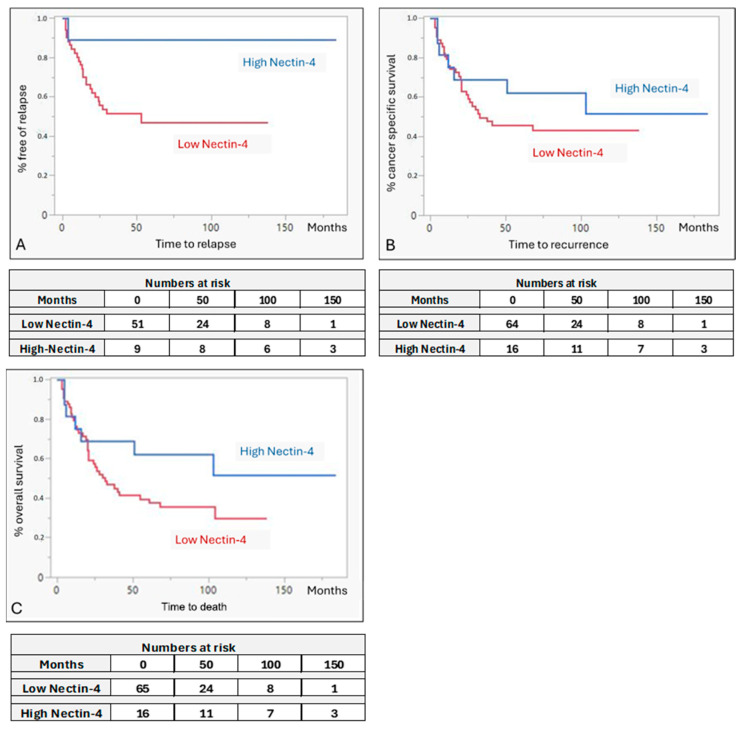
(**A**–**C**): Kaplan–Meier demonstrations of each (**A**): relapse-free—(RFS), (**B**): cancer specific—(CSS) and (**C**): overall survival (OS) for patient groups divided by their tumor tissue Nectin-4 H-score at cut-off 220 of 0–300 possible. Patients with H-scores < 220 suggested having shorter RFS (*p* = 0.0531), CSS (*p* = 0.429) and OS (*p* = 0.1778), respectively.

Survival analyses were performed exclusively in the Discovery Cohort because complete follow-up data required for RFS, CSS, and OS analyses were not available for the Validation Cohort. Relapse-free survival (RFS) analysis (n = 60) indicated a trend toward earlier relapse in patients with Nectin-4 H-scores < 220 (log-rank *p* = 0.0531; Figure 4A). In contrast, no significant associations between Nectin-4 expression and cancer-specific survival (CSS; n = 80) or overall survival (OS; n = 81) were observed (*p* = 0.43 and *p* = 0.18, respectively). Notably, however, all Kaplan–Meier analyses demonstrated a visually less favorable disease course in patients with lower Nectin-4 expression (<220) (Figure 4A–C).

In univariate Cox regression analysis, both T stage and Nectin-4 expression were associated with relapse risk; however, neither retained statistical significance in multivariate analysis. Age, T stage, lymph node status, metastatic status, and tumor grade were significant predictors of CSS and OS in univariate analyses, with patient age emerging as an independent prognostic factor in multivariate models (Table 2).

### 2.6. Correlations with Other Oncologic or Inflammatory Biomarkers

**Figure 5:**
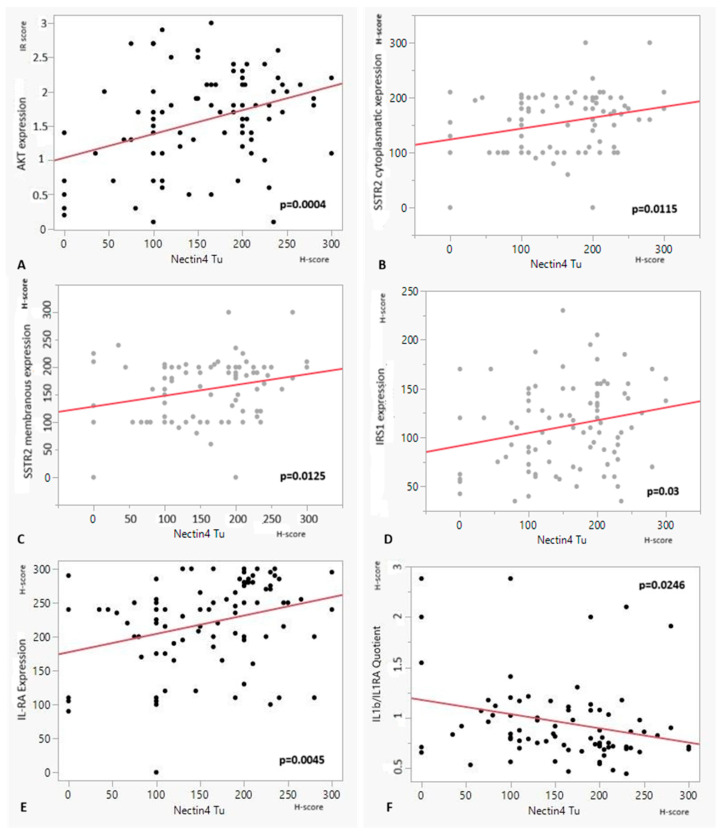
(**A**–**F**): Correlations between Nectin-4 and (**A**): Protein kinase b (Akt), (**B**): Cytoplasmatic- and (**C**): Membranous Somatostatin receptor 2 (SSTR2), (**D**): Insulin receptor substrate 1 (IRS1), (**E**): Interleukin receptor antagonist (IL-RA) expressions and (**F**): The IL-1ß/Interleukin receptor antagonist (IL1ß/IL-RA) ratio according to Vukovic et al. [10] in bladder cancer tissue. Scatter plots show significant correlations with all these parameters in bladder cancer tissue with Akt (*p* = 0.0004), SSTR2 cytoplasmatic (*p* = 0.0115), membranous (*p* = 0.0125), IRS1 (*p* = 0.03), IL-RA (*p* = 0.045) and IL-1ß/IL-RA quotient (*p* = 0.0246). Correlations were positive in all comparisons except with the IL1ß/IL-RA, which showed a negative correspondence.

Significant positive correlations were identified between Nectin-4 expression in bladder cancer tissue and corresponding Akt expression (*p* = 0.0004; Figure 5A), as well as both cytoplasmic and membranous expression of somatostatin receptor 2 (SSTR2; *p* = 0.0115 and *p* = 0.0125, respectively; Figure 5B,C). In addition, Nectin-4 expression positively correlated with insulin receptor substrate 1 (IRS1; *p* = 0.03; Figure 5D) and interleukin-1 receptor antagonist (IL-1RA; *p* = 0.0045; Figure 5E) expression. Furthermore, a significant association was observed with the IL-1β/IL-1RA ratio in urothelial carcinoma tissue (*p* = 0.0246; Figure 5F).

## 3. Discussion

Our study confirms that Nectin-4 is frequently expressed in bladder cancer tissues. Notably, Nectin-4 expression was also observed in histologically benign urothelium adjacent to tumor sites, supporting previous evidence that molecular alterations may precede overt histopathological changes within the urothelium [11,12]. This observation is consistent with the concept of a “field effect” or “field cancerization” in bladder carcinogenesis [11]. The expression distributions demonstrate consistent Nectin-4 expression patterns across both cohorts, with comparable ranges and quartile distributions. These data indicate a broadly similar expression landscape between cohorts, supporting internal consistency of the findings. Importantly, while Nectin-4 expression spans a wide dynamic range, most cases cluster within low to intermediate expression levels, with a smaller subset demonstrating high expression. This distributional pattern provides the basis for the exploratory stratification into high versus low expression groups and supports the biological interpretation that only a subset of tumors exhibits strong Nectin-4 upregulation. Although no independent prognostic value was observed, these expression patterns support the concept that Nectin-4 reflects heterogeneous biological activity across MIBC tumors, consistent with its role as part of broader tumor-associated signaling networks.

Contrary to our initial expectations, no statistically significant associations were identified between Nectin-4 expression and major clinicopathological parameters in MIBC, including tumor stage and grade. Nevertheless, the observed trend toward lower Nectin-4 expression in higher-grade and more advanced tumors is consistent with the hypothesis that adhesion molecules such as nectins may become downregulated during tumor progression and invasion [13,14]. Such downregulation may facilitate tumor cell detachment and metastatic dissemination, a phenomenon previously described in several other malignancies [11]. These findings further complement previous concepts of generalized Nectin-4 overexpression in MIBC by suggesting a more patient-specific and potentially stage-dependent biological relevance [9].

The observed positive correlations between Nectin-4 expression and Akt, SSTR2, IRS1, as well as the IL-1β/IL-1RA system, suggest a multifaceted role of Nectin-4 in tumor biology involving cell signaling, metabolic regulation, and tumor-associated immune interactions. The PI3K/AKT pathway, represented by Akt, is a well-established therapeutic axis in multiple solid tumors, with ongoing preclinical and early-phase clinical studies investigating pathway inhibition alone or in combination with systemic therapies [10]. In contrast, IRS1, as part of the insulin/IGF signaling network, has been implicated in tumor progression and metabolic reprogramming, but remains an investigational target without established clinical translation. Somatostatin receptor 2 (SSTR2) has been implicated in tumor growth inhibition and immune modulation [15]. It represents a clinically validated target in other tumor entities, where receptor-directed imaging and peptide receptor radionuclide therapy have demonstrated efficacy, suggesting potential translational relevance in SSTR2-expressing tumors, although its role in bladder cancer remains undefined. The correlation with insulin receptor substrate 1 (IRS1), a key mediator of insulin and IGF-1 signaling pathways, may point to links between Nectin-4 and metabolic pathways that promote tumor cell survival [15]. Notably, the association with IL-1β, a pro-inflammatory cytokine, hints at an involvement of Nectin-4 in modulating the tumor microenvironment and immune responses [14]. Elevated IL-1β expression has been linked to both tumor progression and anti-tumor immunity depending on context [16]. Additionally, our previous study demonstrated that the IL-1β/IL-1RA axis reflects inflammatory signaling within the tumor microenvironment, which is increasingly recognized as a regulator of tumor progression and therapeutic response, although it remains an investigational therapeutic target in urothelial carcinoma [10]. The observed associations between high Nectin-4 expression and markers of growth signaling, metabolic regulation, and inflammation suggest that Nectin-4 may function as part of a broader tumor-promoting network rather than as an isolated surface antigen. Importantly, these pathways are not established therapeutic targets in bladder cancer. Their association with Nectin-4 expression is therefore hypothesis-generating and supports the concept that Nectin-4 is embedded within a broader tumor-associated signaling network that may inform future combination strategies.

Although survival analyses did not demonstrate statistically significant prognostic value for Nectin-4 expression, the observed trend toward improved relapse-free survival in patients with higher Nectin-4 levels warrants further investigation. Larger prospective and multi-institutional studies are needed to validate these findings. This is particularly relevant given that multivariate analysis confirmed established prognostic factors, including patient age, tumor stage, and lymph node status, as independent predictors of outcome, consistent with previous reports [17].

Moreover, accumulating evidence suggests that Nectin-4 expression may have prognostic relevance in relation to disease progression, as expression is frequently reduced or absent in metastatic urothelial carcinoma tissue [9]. Importantly, the clinical efficacy of Enfortumab Vedotin is closely linked to membranous Nectin-4 expression, highlighting its role not only as a therapeutic target but also as a potential predictive biomarker [18]. In light of the increasing implementation of neoadjuvant and perioperative systemic therapies in MIBC, the distribution and biological behavior of Nectin-4 expression in pretreated compared with treatment-naïve tumors warrant particular attention. Although our study was not designed to evaluate treatment response, the present findings raise the hypothesis that Nectin-4-associated signaling and inflammatory pathways may either influence, or be modulated by, exposure to neoadjuvant therapy.

Taken together, our findings support the need for further translational research aimed at clarifying the biological and prognostic significance of Nectin-4 in bladder cancer. A more comprehensive understanding of its interactions with inflammatory, immune-related, and growth-associated signaling pathways may improve current biomarker strategies and refine the predictive role of Nectin-4 in therapeutic response.

Several limitations of this study should be acknowledged. First, the retrospective study design introduces the potential for selection bias. Second, the relatively limited number of patients with complete survival data reduced the statistical power of survival analyses. Finally, the semi-quantitative nature of immunohistochemical H-scoring may introduce interobserver variability, underscoring the importance of standardized assessment protocols and automated quantitative approaches in future studies. We acknowledge that multiple correlation analyses were performed without adjustment for multiple comparisons. This approach was chosen due to the exploratory nature of the study and the limited sample size, in order to avoid excessive reduction in statistical power. However, this increases the risk of type I error, and therefore the observed associations, particularly those with borderline significance, should be interpreted as hypothesis-generating and require validation in independent cohorts. An additional important consideration is the heterogeneity of scoring approaches used across studies. While several prognostic and predictive analyses have emphasized membranous Nectin-4 expression as biologically and clinically relevant [9], in the present study Nectin-4 expression was assessed using a combined H-score without separate evaluation of membranous and cytoplasmic staining. Given the established clinical relevance of membranous Nectin-4 in antibody–drug conjugate therapy, future studies should differentiate subcellular localization to better define its biological and clinical significance. Accordingly, the correlations identified in the present study reflect overall Nectin-4 immunoreactivity rather than therapeutically relevant membranous target availability. Therefore, these findings should not be directly extrapolated to prediction of response to Enfortumab Vedotin and should be interpreted primarily in a biological and hypothesis-generating context. Although modeling Nectin-4 as a continuous variable is statistically preferable, this approach was not used as the primary analytical strategy in the present study due to the focus on immunohistochemical stratification and the limited number of outcome events. Secondary analyses of pivotal antibody-drug conjugate trials have frequently relied on broader composite or overall staining scores. The present study contributes to this ongoing discussion by highlighting how different assessment strategies may capture distinct biological aspects of Nectin-4 expression. It is important to emphasize that the associations identified in this study are based on immunohistochemical correlation analyses and do not imply causal or functional relationships between Nectin-4 and the investigated signaling pathways. In addition, no external validation using transcriptomic or proteomic datasets (e.g., TCGA) was performed. Therefore, these findings should be interpreted as hypothesis-generating and require validation in independent cohorts as well as functional experimental models.

In summary, although Nectin-4 expression alone does not appear to represent a robust independent prognostic biomarker, our findings support the concept that Nectin-4 expression in MIBC reflects an integrated biological program with potential implications for patient stratification and the development of rational perioperative combination therapies. Its frequent expression and association with key tumor-related signaling molecules further underscore its relevance as a candidate for translational investigation. Future studies exploring its mechanistic role and therapeutic exploitation—potentially in combination with pathways involving SSTR2 and IL-1β—may contribute to more personalized treatment strategies for patients with bladder cancer.

## 4. Materials and Methods

### 4.1. Patient Cohorts

The study was approved by the Ethics Committee of the Medical Faculty of the Eberhard Karls University Tübingen following formal review and evaluation (project no. 279/2013BO2; approved 1 March 2023). Two independent retrospective cohorts of patients who underwent radical cystectomy for invasive bladder cancer (BC) at the University Hospital Tübingen were analyzed.

The discovery cohort comprised 103 patients treated between 1996 and 2006, whereas the validation cohort included 97 patients fulfilling the same inclusion criteria. Eligible patients had pathological tumor stage pT1 or higher. All tissue samples were obtained from cystectomy specimens.

For tissue microarray (TMA) construction, representative invasive tumor regions were selected by an experienced uropathologist based on hematoxylin and eosin-stained whole-slide sections. Areas containing viable and morphologically representative tumor tissue were preferentially chosen, while necrotic and artifact-containing regions were avoided. For each tumor, 2–3 tissue cores (1.0 mm diameter) were sampled from multiple representative tumor regions and incorporated into the TMA. Accordingly, immunohistochemical analysis was performed on multiple tumor regions per case to account for potential intratumoral heterogeneity. This approach was applied to improve representativeness and reduce sampling bias due to intratumoral heterogeneity. Although conducted at a single institution, the study population was divided into two cohorts to enable internal comparison and exploratory validation of the observed associations. Furthermore, correlations with inflammatory and oncologic biomarkers were evaluated exclusively in the first cohort. This approach was employed to assess the robustness and consistency of Nectin-4-related findings across clinically comparable yet distinct patient subsets. Long-term follow-up data required for survival analyses were available only in the Discovery Cohort; therefore, Kaplan–Meier and Cox regression analyses were restricted to this cohort.

### 4.2. Immunohistochemistry

Sections from tissue microarrays (TMAs), constructed using a Beecher MTA-1 manual tissue arrayer (Beecher Instruments, Inc., Sun Prairie, WI, USA), were stained for Nectin-4 and the four aforementioned markers using an indirect immunohistochemical method in the Department of Urology’s clinical laboratory. For Nectin-4 immunostaining, a polyclonal rabbit IgG antibody (PA5-50463; Thermo Fisher Scientific, Waltham, MA, USA) was applied at a dilution of 1:2000 using the ZytoChem HRP One-Step Polymer anti-rabbit/mouse detection kit (Zytomed, Berlin, Germany). Visualization was performed with Dako Liquid DAB chromogen (Dako, Glostrup, Denmark; now Agilent Technologies, Santa Clara, CA, USA), followed by counterstaining with Mayer’s hematoxylin.

Immunohistochemical staining for protein kinase B (Akt) and IL-1β was carried out as previously described by Vukovic et al. [10]. Akt expression was detected using the monoclonal mouse pan-AKT antibody (40D4; Cell Signaling Technology, Danvers, MA, USA) at a dilution of 1:800 in combination with the Dako REAL™ Rabbit/Mouse Avidin-Biotin Detection System including Peroxidase/DAB+ (Dako). For IL-1β and IL-1 receptor antagonist (IL-1F3), polyclonal goat IgG antibodies (AF-201 and AF-280; R&D Systems, Minneapolis, MN, USA) were used at dilutions of 1:10 and 1:300, respectively, together with the Biozol HRP One-Step Polymer anti-goat detection kit (BIOZOL Diagnostica, Hamburg, Germany). Visualization was performed using the Dako Liquid DAB Substrate Chromogen System K3467 (Dako) [10].

Somatostatin receptor 2 (SST2) staining was performed as previously reported [19] using the rabbit monoclonal anti-somatostatin receptor 2 antibody UMB1 (ab134152; Abcam, Cambridge, UK) at a dilution of 1:200 in combination with the Dako REAL™ Peroxidase/DAB+ Rabbit/Mouse Detection System (Dako). Insulin receptor substrate 1 (IRS1) expression was detected using a rabbit anti-IRS1 antibody (C-20; sc-559; Santa Cruz Biotechnology, Dallas, TX, USA) at a dilution of 1:400 and visualized using the VECTASTAIN Elite ABC Kit (Vector Laboratories, Newark, CA, USA) with DAB substrate (SK-4100; Vector Laboratories).

**Figure 6:**
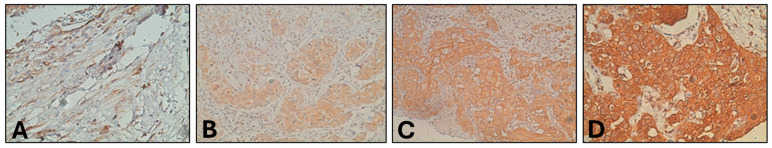
(**A**–**D**): Nectin-4 Scaling of urothelial carcinoma tissue in TMA’s with (**A**): 0—no staining, (**B**): 1—low staining, (**C**): 2—medium staining and (**D**): 3—strong staining (200× magnification each).

**Figure 7:**
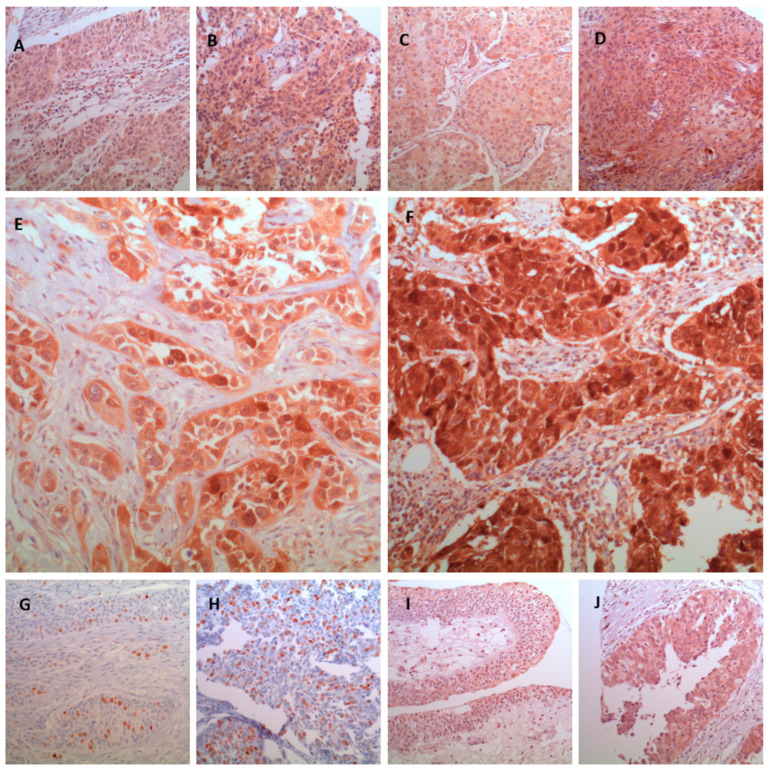
(**A**–**J**): Representative staining for immunohistochemistry of IL-1β/IL-1RA, AKT, IRS1 and SST2 signaling pathway markers in bladder cancer and normal tissues. (**A**,**B**) Cytoplasmic IRS1 immunostaining in bladder tissue: (**A**)—staining in benign tissue; (**B**)—high staining in cancer tissue. (**C**,**D**) Cytoplasmic IL-1RA immunostaining in bladder tissue: (**C**)—staining in benign tissue; (**D**)—high staining in cancer tissue. (**E**,**F**) Cytoplasmic and nuclear SST2 staining in bladder tissue: (**E**)—staining in benign tissue; (**F**)—high staining in cancer tissue; (**G**,**H**) Cytoplasmic and partial nuclear AKT staining in bladder tissue: (**G**)—staining in benign tissue; (**F**)—high (>15%) staining in cancer tissue; (**I**,**J**) Cytoplasmic IL-1 β immunostaining in bladder tissue: (**C**)—staining in benign tissue; (**D**)—high staining in cancer tissue. Magnification: 200-fold.

Protein expression was quantified using the H-score method, calculated as the product of the percentage of positively stained cells and staining intensity, with intensity graded from 0 (no staining) to 3 (strong staining) [20]. In cases with multiple tumor cores, staining results were averaged to generate a final H-score for each patient. Thereby, all urothelial tumor cells or all benign urothelium cells within one dot were set as 100% (1.0 in case of Akt). This resulted in numeric demonstrations of each expression 0–300 (0–3 in case of Akt). Immunohistochemical images were captured by ProgRes Capture Pro 2.10.0.1—Jenoptic (Jena, Germany). Reference images for each intensity (0–3) were created and applied during evaluation (Figure 6). Optical evaluation was conducted with a Carl Zeiss Axioskope at 25×, 160×, and 400× magnifications, blinded to clinical data (Figure 6 and Figure 7). Nectin-4 expression was assessed using a combined H-score integrating staining intensity across cellular compartments, without separate compartment-specific scoring of membranous and cytoplasmic expression.

### 4.3. Statistical Analysis

Because quantile–quantile plot analysis indicated that the data were not normally distributed, non-parametric statistical methods were applied. Differences in H-scores between groups were assessed using the Wilcoxon rank-sum test or the Kruskal–Wallis test, as appropriate. Likewise, associations between Nectin-4 expression and clinicopathological parameters were analyzed using non-parametric approaches.

Survival analyses, including relapse-free survival (RFS), cancer-specific survival (CSS), and overall survival (OS), were performed using the log-rank test and visualized by Kaplan–Meier curves. Univariate and multivariate Cox proportional hazards regression models were used to evaluate the independent prognostic significance of the analyzed variables.

Correlations between Nectin-4 expression and oncological or immunological biomarkers were assessed by linear regression analysis. A two-sided *p*-value < 0.05 was considered statistically significant. Statistical analyses were performed using JMP software version 16 (SAS Institute Inc., Cary, NC, USA).

Given the exploratory nature of the study and the limited sample size, no adjustment for multiple testing was applied. Therefore, all correlation analyses should be interpreted as hypothesis-generating. The cutoff value (H-score 220) was data-driven and derived within the study cohort**.** Specifically, it was defined based on statistical distribution of Nectin-4 expression values in the combined cohort, using the median/optimal separation of the dataset to explore potential associations with clinical outcomes in an exploratory manner. This threshold was not pre-specified or externally validated and is therefore interpreted in a hypothesis-generating context.

## 5. Conclusions

Our findings indicate that high Nectin-4 expression in muscle-invasive bladder cancer is associated with a complex network of growth-promoting and inflammatory signaling pathways. This integrated molecular profile may contribute to the biological and clinical relevance of Nectin-4-targeted therapies and provides a rationale for exploring combination treatment strategies directed against both Nectin-4 and its associated downstream pathways.

Future studies should investigate whether these molecular characteristics may support patient stratification and treatment optimization in the neoadjuvant and perioperative setting. Prospective validation in larger, independent cohorts will be necessary to further define the predictive and clinical significance of these observations.

## Figures and Tables

**Table 1:** Patients’ characteristics.

Discovery Group Median n %				Validation Cohort Median n %				*p*
Age	65.9	103		Age	64.4	97		0.72
Gender (n = 103)				Gender (n = 97)				0.56
Male Female		79 24	77.7 23.3	Male Female		70 27	72.2 27.8	
T-Status (n = 103)	T3a			T-Status (n = 97)	T3a			0.53
T1 T2a T2b T3a T3b T4a T4b		1 16 14 24 24 18 6	0.9 15.6 13.6 23.3 23.3 17.5 5.8	T1 T2a T2b T3a T3b T4a T4b		7 16 14 22 22 12 4	7.2 16.5 14.5 22.6 22.6 12.4 4.1	
N-Status (n = 99)	0			N-Status (n = 94)	0			0.41
N0 N1 N2 N3		58 24 15 2	58.2 24.5 15.3 2.0	N0 N+		61 33 - -	64.8 35.2 - -	
M-Status (n = 99)	0			M-Status (n = 99)	0			0.77
M0 M1		90 9	90.1 9.9	M0 M1		86 11	88.6 11.4	
Grading (n = 103)	3			Grading (n = 96)	3			0.84
G1 G2 G3		025 78	23.5 76.5	G1 G2 G3		1 19 76	1 19.7 79.3	

**Table 2:** Univariate and multivariate Cox regression analyses for recurrence-free survival (RFS), cancer-specific survival (CSS), and overall survival (OS) in the Discovery Cohort.

Variable	Univariate Analysis			Multivariate Analysis		
RFS	*p*	Hazard ratio	95% CI	*p*	Hazard ratio	95%CI
Pathological stage (T > 2 vs. T2)	0.0099	3.25	1.33–7.95	0.16	1.92	0.77–4.81
Nodal status (N+ vs. N0)	0.0879	1.88	0.91–3.88	-	-	-
Metastasis status (M+ vs. M0)		-	-	-	-	-
Grade (≥G3 vs. <G3)	0.0531	1.30	0.60–2.83	-	-	-
Nectin 4 (low vs. high tumor expression) (cut-off 220)	0.09	5.66	0.77–41.79	0.13	4.61	0.61–34.57
Age, year	0.2397	1.02	0.99–1.06	-	-	-
CSS	**Univariate analysis**			**Multivariate analysis**		
	*p*	Hazard ratio	95% CI	*p*	Hazard ratio	95%CI
Pathological stage (T > 2 vs. T2)	0.0107	2.53	1.24–5.17	0.25	1.59	0.72–3.51
Nodal status (N+ vs. N0)	0.0309	1.96	1.06–3.62	0.21	1.60	0.76–3.33
Metastasis status (M+ vs. M0)	0.0015	3.56	1.63–7.79	0.11	2.16	0.85–5.50
Grade (G ≥ 3 vs. G < 3)	0.0069	3.62	1.42–9.22	0.26	1.78	0.65–4.88
Nectin 4 (low vs. high tumor expression) (cut-off 220)	0.43	1.39	0.61–3.17	0.91	1.05	0.44–2.51
Age, year	0.0047	1.05	1.01–1.08	0.0423	1.04	1.00–1.07
OS	**Univariate analysis**			**Multivariate analysis**		
	*p*	Hazard ratio	95% CI	*p*	Hazard ratio	95%CI
Pathological stage (T > 2 vs. T2)	0.0020	2.79	1.45–5.33	0.15	1.74	0.83–3.65
Nodal status (N+ vs. N0)	0.0258	1.85	1.08–3.17	0.0673	1.92	0.95–3.84
Metastasis status (M+ vs. M0)	0.0036	3.13	1.45–6.73	0.21	1.81	0.71–4.58
Grade (G ≥ 3 vs. <G3)	0.0173	2.31	1.16–4.60	0.41	1.45	0.60–3.46
Nectin 4 (low vs. high tumor expression) (cut-off 220)	0.19	1.73	0.77–3.89	0.47	1.37	0.58–3.27
Age, year	0.0031	1.05	1.02–1.08	0.0191	1.04	1.01–1.08

## Data Availability

The data from this study are available by request from the corresponding author. The data are not publicly available due to ethical restrictions (IRB statement).
